# Oxidized mtDNA Contributes to Pulmonary Inflammation and Fibrosis in Bleomycin‐Induced Lung Injury

**DOI:** 10.1002/mco2.70664

**Published:** 2026-04-02

**Authors:** Ye Mao, Xinyu Tian, Jiayuan Ai, Xiaoting Zhou, Yanghong Ni, Dandan Wan, Min Luo, Xiawei Wei

**Affiliations:** ^1^ Laboratory of Aging Research and Cancer Drug Target, State Key Laboratory of Biotherapy, National Clinical Research Centre for Geriatrics West China Hospital, Sichuan University Chengdu Sichuan P.R. China; ^2^ Department of Oncology The Second Affiliated Hospital of Nanchang University Nanchang China; ^3^ Department of Biotherapy, Laboratory of Aging Research and Cancer Drug Target, State Key Laboratory of Biotherapy, National Clinical Research Center for Geriatrics, West China Hospital, Sichuan University

**Keywords:** bleomycin, inflammation, macrophage polarization, oxidized mitochondrial DNA, pulmonary fibrosis

## Abstract

Pulmonary fibrosis is a chronic and progressive interstitial lung disease with limited treatment options aside from lung transplantation. Bleomycin (BLM)‐induced lung injury is the most commonly used experimental model to mimic the key pathological features of human pulmonary fibrosis, which include an early inflammatory phase and a later fibrotic phase. Neutrophil infiltration and M2 macrophage activation are key events in these stages, respectively. However, the molecular mechanisms by which BLM triggers pulmonary inflammation and fibrosis remain incompletely understood. In this study, we found that BLM treatment induced ROS‐mediated oxidative damage in the lungs, leading to an inflammatory microenvironment and the release of oxidized mitochondrial DNA (oxid‐mtDNA). Oxid‐mtDNA was shown to contribute to the early inflammatory response by promoting neutrophil recruitment and enhancing macrophage polarization, which subsequently drove tissue remodeling and fibrosis. Notably, direct injection of oxid‐mtDNA into the lungs recapitulated the fibrotic features observed in the BLM model. Furthermore, studies using STING‐ and NLRP3‐deficient mice demonstrated that loss of either pathway significantly attenuated BLM‐induced inflammation and fibrosis, implicating their involvement downstream of oxid‐mtDNA signaling. Collectively, our findings identify oxid‐mtDNA as a critical mediator linking oxidative injury to immune activation and fibrotic remodeling in the lung, offering new insights into pulmonary fibrosis pathogenesis and potential therapeutic targets.

## Graphical abstract

In this study, we demonstrate that bleomycin induces the release of reactive oxygen species (ROS), resulting in oxidative damage to mitochondrial DNA (mtDNA), which subsequently triggers neutrophil infiltration and M2 macrophage polarization in the lungs of mice. These events ultimately contribute to the development of pulmonary fibrosis. Additionally, we provide evidence that the bleomycin‐induced fibrosis process is closely associated with the activation of the STING and NLRP3 signaling pathways.

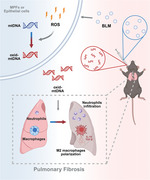

## 1 Introduction

Pulmonary fibrosis is a chronic, progressive, and often fatal interstitial lung disease characterized by excessive deposition of extracellular matrix and the formation of fibrotic scars, leading to irreversible structural remodeling and functional impairment of the lungs [1, 2, 3, 4]. Current therapeutic strategies for pulmonary fibrosis primarily include pharmacological interventions such as pirfenidone and nintedanib, supplemental oxygen therapy, pulmonary rehabilitation, and lung transplantation. However, these treatments offer only limited efficacy and are unable to halt or reverse fibrotic progression [5, 6].

Bleomycin (BLM) is widely employed via intratracheal injection to induce pulmonary fibrosis in animal models [7, 8, 9, 10, 11]. This model effectively replicates several pathological features of human idiopathic pulmonary fibrosis (IPF), including alveolar epithelial cell injury, inflammatory responses, fibroblast activation, and collagen deposition. Intratracheal injection of BLM induces an inflammatory response within 5–7 days, which subsequently progresses to fibrosis within 3–4 weeks. Consequently, the BLM‐induced pulmonary fibrosis model is extensively utilized for evaluating the anti‐fibrotic effects of various drugs or therapeutic approaches.

Previous studies have demonstrated that bleomycin (BLM) induces pulmonary fibrosis through cytotoxic effects on alveolar epithelial cells, triggering apoptosis or necrosis and thereby initiating the fibrotic cascade [12, 13, 14]. Injured epithelial cells release various pro‐inflammatory cytokines, such as interleukin‐6 (IL‐6), which promote the recruitment of macrophages and neutrophils into the affected regions of the lung [15, 16, 17]. These infiltrating immune cells further secrete profibrotic mediators, including transforming growth factor‐beta (TGF‐β), which in turn activate and promote the proliferation of fibroblasts [18, 19]. The resultant fibroblast activation leads to excessive collagen deposition and destruction of normal lung architecture. However, the mechanism by which BLM induces pulmonary fibrosis has not been fully elucidated. Therefore, in this article, we further discuss the underlying mechanism of the BLM‐induced pulmonary fibrosis model, which is the most commonly used animal model displaying both inflammatory and fibrotic progression.

Various inflammatory cells are involved in BLM‐induced pulmonary fibrosis, including neutrophils and macrophages. In the early phase, neutrophils predominate and contribute to lung injury by releasing pro‐inflammatory cytokines such as interleukin‐6 (IL‐6) and reactive oxygen species (ROS), thereby promoting epithelial damage and establishing a profibrotic microenvironment [4, 10, 20, 21, 22]. This inflammatory milieu facilitates the release of profibrotic mediators, such as TGF‐β, further driving fibrosis [23, 24, 25]. In the fibrotic phase, M2 macrophages become the primary source of profibrotic mediators such as TGF‐β and IL‐10, promoting fibroblast activation and accelerating collagen deposition and tissue remodeling [11, 26, 27, 28, 29, 30]. Their excessive activation, along with crosstalk with neutrophils, forms a vicious cycle that sustains fibrosis, partly via the stimulator of interferon genes (STING) and NOD‐like receptor family, pyrin domain containing 3 (NLRP3) signaling pathways [31, 32].

In this study, we employed a BLM‐induced pulmonary inflammation and fibrosis model to demonstrate that BLM enhances ROS generation, triggering oxidative stress and inducing mitochondrial DNA (mtDNA) damage. The resulting oxidized mtDNA (oxid‐mtDNA) drives M2 macrophage polarization and the secretion of profibrotic cytokines, ultimately promoting pulmonary fibrosis. Mechanistically, we identified that this process is critically mediated by the STING and NLRP3 signaling pathways. Oxid‐mtDNA activates both pathways in macrophages, whereas genetic deficiency of either STING or NLRP3 confers protection against bleomycin‐induced pulmonary fibrosis. These key findings contribute to a better understanding of the progression mechanisms of pulmonary fibrosis and facilitate the development of novel therapies targeting oxidative stress in pulmonary fibrosis.

## 2 Materials and Methods

### 2.1 Mice

Male C57BL/6 wild‐type mice were purchased from Beijing Vital River Laboratory Animal Technology Company (6–8 weeks old, weighing 19–21 g). C57BL/6 mice expressing NLRP3^−/−^ were purchased from Genetech, USA. C57BL/6 mice expressing Tmem173^−/−^ (STING knockout) were purchased from The Jackson Laboratory (Bar Harbor, ME, USA). The mice were housed and maintained under specific pathogen‐free (SPF) conditions in an animal facility under a 12 h light/ 12 h dark cycle at 25°C. All animal experiments were performed according to protocols approved by the Animal Care and Use Committee of Sichuan University (Chengdu, Sichuan, China).

### 2.2 BLM‐Induced Lung Inflammation and Pulmonary Fibrosis Model

To establish the BLM‐induced lung inflammation and pulmonary fibrosis model, male mice (8 weeks old) were anesthetized by intraperitoneal injection of 25 µg/mL tribromoethanol, 200 µL/each. After being fully anesthetized, the neck of the mice was disinfected with iodophor, and the trachea was exposed with sterilized surgical instruments. Then, a single injection of BLM (50 µL, 2 mg/kg) was administered via intratracheal instillation as previously described [11]. The lung tissues from the injected mice were collected on 1, 3, 5, 7, and 21 days post‐treatment and were investigated for pathological changes. For NAC treatment, 30 min before the intratracheal injection of BLM, mice in the BLM+NAC group or NAC group were intraperitoneally injected with NAC (200 µL, 100 mg/kg). Lung tissues were collected 24 h later.

### 2.3 Cell Culture

For mouse pulmonary fibroblasts (MPF) extraction, lung tissues were collected under aseptic conditions and minced into small pieces of approximately 1 mm^3^. These tissue pieces were then placed into collagenase I (Sigma) in Rowell Park Memorial Institute‐1640 medium (RPMI‐1640 medium) (Gibco‐Invitrogen) at 37°C for 2 h. Then the cell suspension was centrifuged, washed twice with RPMI‐1640 medium, and cultured in RPMI‐1640 medium with 10% fetal bovine serum (FBS) (Gibco‐Invitrogen).

For peritoneal macrophage extraction, mice were euthanized, and 10 mL of saline was injected intraperitoneally, followed by a 30‐s pause to allow sufficient contact before slowly withdrawing the intraperitoneal lavage liquid. The cells were centrifuged, resuspended in RPIM‐1640 medium, and the process was repeated once. The cells were then plated in 6‐well plates and cultured in RPMI 1640 medium containing 10% FBS, penicillin and streptomycin at 37°C for 2 h. Adherent cells were collected as peritoneal macrophages.

To collect bone marrow‐derived macrophages (BMDMs), femur bone cavities of C57BL/6 mice (6–8 weeks) were flushed using saline with a 1 mL syringe. The cells were plated in 6‐well plates containing DMEM supplemented with 20 ng/mL of mouse macrophage colony‐stimulating factor (M‐CSF) (R&D Systems and PeproTech). Three days later, the supernatant was discarded, and the adherent cells were cultured for 7 days before further experiments. For siRNA silencing, on Day 3 of BMDM culture, the existing medium was removed and replaced with Opti‐MEM (Gibco) containing diluted siRNA and Lipofectamine RNAiMAX Transfection Reagent (Thermo Fisher) according to the manufacturer's instructions. After 24 h of incubation, the transfection medium was replaced with fresh complete medium. Cells and supernatants were collected 48 h post‐transfection for further use.

The sequences of siRNA used were as follows:

si*Sting1*‐1: sense, 5’‐ GCUAUGAUUCUACUAUCGU(dT)(dT) ‐3’
si*Sting1*‐1: antisense, 5’‐ ACGAUAGUAGAAUCAUAGC(dT)(dT) ‐3’
si*Sting1*‐2: sense, 5’‐ GGUACUUGCGGUUGAUCUU(dT)(dT) ‐3’
si*Sting1*‐2: antisense, 5’‐ AAGAUCAACCGCAAGUACC(dT)(dT) ‐3’
si*Sting1*‐3: sense, 5’‐ CAAAGGAUCCACCAAAUCA(dT)(dT) ‐3’
si*Sting1*‐3: antisense, 5’‐ UGAUUUGGUGGAUCCUUUG(dT)x02010;3’
si*Nlrp3*‐1: sense, 5’‐ GGAUCUUUGCUGCGAUCAA(dT)(dT)10;3’
si*Nlrp3*‐1: antisense, 5’‐ UUGAUCGCAGCAAAGAUCC(dT)(dT) ‐3’
si*Nlrp3*‐2: sense, 5’‐ CGUGAAGGUCCUACUAGAA(dT)(dT) ‐3’
si*Nlrp3*‐2: antisense, 5’‐ UUCUAGUAGGACCUUCACG(dT)(dT) ‐3’
si*Nlrp3*‐3: sense, 5’‐ GCAGGUUCUACUCUAUCAA(dT)(dT) ‐3’
si*Nlrp3*‐3: antisense, 5’‐ UUGAUAGAGUAGAACCUGC(dT)(dT) ‐3’

### 2.4 Bronchoalveolar Lavage Fluid (BALF) Collection

To collect BALF, a precise incision was made along the tracheal cartilage ring of the anesthetized mouse. Gently insert the needle into the trachea and slowly inject 1 mL of sterile saline solution into the lung. After allowing a 30‐s pause to ensure sufficient contact, slowly withdraw the needle. Repeat this process three times to collect BALF. The collected BALF was kept on ice for further use.

### 2.5 Reactive Oxygen Species (ROS) Production Measurement

To assess total ROS production, MPFs were treated with 20 µg/mL BLM for 5 min and then washed twice with PBS. Then the cells were stained with 10 µM cell‐permeable fluorescent 2′, 7′‐dichlorofluorescin diacetates (H2DCF‐DA) (Sigma) at 37°C for 30 min, and the fluorescence intensity was analyzed using the NovoCyte Flow Cytometer (ACEA Biosciences).

### 2.6 Flow Cytometry

For inflammatory cells in lung tissue, lung tissues were minced into small pieces of approximately 1 mm^3^ and digested with collagen‐I in RPMI‐1640 basic medium at 37°C for 2 h. The tissue suspension was filtered through a 70 µm nylon mesh and centrifuged. The red blood cell lysis buffer was added, and the suspended cells were centrifuged and washed twice with PBS. Cells were then stained with antibodies for 30 min at 4°C in the dark. Neutrophils were stained with the following antibodies: anti‐CD11b‐FITC (Biolegend, 1:100), anti‐CD45‐PE (Biolegend, 1:100), anti‐Ly6G‐APC (BD Pharmingen, 1:100). Macrophages were stained with the following antibodies: anti‐CD11c‐FITC (Biolegend, 1:100), anti‐CD45‐PE (Biolegend, 1:100), anti‐CD206‐APC (Biolegend, 1:100), anti‐F4/80‐Percp5.5 (Biolegend, 1:100).

For 8‐hydroxy‐2′‐deoxyguanosine (8‐OHdG) quantification, MPFs were cultured with medium, 20 µg/mL BLM, and 20 µg/mL bleomycin with 5 mM N‐acetylcysteine (NAC) for 24 h. The cells were then digested, centrifuged, and resuspended in PBS. The cells were then fixed with 2% paraformaldehyde for 30 min at room temperature and incubated at 4°C overnight with 1% Triton X‐100 containing anti‐8‐OHdG antibody (Novus, 1:300). Subsequently, the cells were incubated with goat anti‐rabbit‐FITC secondary antibody (Life Technologies, 1:1000) in the dark at a low temperature for 30 min. Finally, the cells were washed twice with PBS and detected on NovoCyte Flow Cytometer (ACEA Biosciences, Inc., San Diego, CA, USA). Data were analyzed using NovoExpress software (1.3.0, ACEA Biosciences, Inc., San Diego, CA, USA, 2018).

### 2.7 Mito Tracker Determination for Cellular Mitochondria Release

MPFs were cultured in RPMI‐1640 medium with 20 µg/mL BLM for 24 h. Cells were then stained with MitoTracker Red CM‐H2Xros (20 nM) for 30 min at 37°C in the dark and washed twice, followed by DiD (Beyotime, C1995S) staining for 20 min at 37°C. Finally, MPFs were stained with Hoechst 33342 for 10 min at room temperature in the dark, washed twice, and fixed with 4% paraformaldehyde for 15 min at 37°C. The images were captured using a Leica DM6 B Positive Binocular Biomicroscope (Leica).

### 2.8 Quantitative Real‐Time PCR

mtDNA in BALF was extracted and concentrated using the QIAamp DNA Blood Mini Kit (Qiagen). Quantification was performed by quantitative real‐time PCR using TaqMan probes (Thermo Fisher Scientific) and SYBR Green Supermix (Bio‐Rad) on a CFX Connect Real‐Time PCR Detection System (Bio‐Rad). The specific primer and probe sequences used were as follows:

Forward primer, 5’‐ACCTACCCTATCACTCACACTAGCA‐3’,
reverse primer, 5’‐GAGGCTCATCCTGATCATAGAATG‐3’,
probe, ATGAGTTCCCCTACCAATACCACACCC.

For qRT‐PCR of BMDMs, total RNA was extracted using a Cell Total RNA Isolation Kit (Foregene). Quantification was performed by quantitative real‐time PCR using SYBR Green Supermix (Bio‐Rad) on a CFX Connect Real‐Time PCR Detection System (Bio‐Rad). The specific primer and probe sequences used were as follows:

*Gapdh* Forward primer, 5’‐ CAACAGCAACTCCCACTCTTCCA ‐3’
*Gapdh* Reverse primer, 5’‐ ACCCTGTTGCTGTAGCCGTAT ‐3’
*Mrc1* Forward primer, 5’‐ CTCTGTTCAGCTATTGGACGC ‐3’
*Mrc1* Reverse primer, 5’‐ TGGCACTCCCAAACATAATTTGA ‐3’
*Arg1* Forward primer, 5’‐ GTACATTGGCTTGCGAGACG ‐3’
*Arg1* Reverse primer, 5’‐ CGGCCTTTTCTTCCTTCC ‐3’
*Ifnb1* Forward primer, 5’‐ CTGGCTTCCATCATGAACAA ‐3’
*Ifnb1* Reverse primer, 5’‐ AGAGGGCTGTGGTGGAGAA ‐3’
*Sting1* Forward primer, 5’‐ TCCAGGAACACCGGTCTAGG ‐3’
*Sting1* Reversed primer, 5’‐ TCCGTCTGTGGGTTCTTGGT ‐3’
*Nlrp3* Forward primer, 5’‐ TCACAACTCGCCCAAGGAGGAA ‐3’
*Nlrp3* Reversed primer, 5’‐ AAGAGACCACGGCAGAAGCTAG ‐3’
*Il18* Forward primer, 5’‐ GACAGCCTGTGTTCGAGGATATG ‐3’
*Il18* Reversed primer, 5’‐ TGTTCTTACAGGAGAGGGTAGAC ‐3’

### 2.9 Immunofluorescence and Histological Examination

For samples of mouse lung, fresh lung tissue was fixed with 4% paraformaldehyde, embedded in paraffin, and sectioned into 4‐µm‐thick sections and subjected to an EDTA buffer for antigen retrieval. Then the samples were permeabilized with 1% Triton X‐100 for 30 min and incubated with 10% goat serum for blocking. For samples from cultured cells, the MPFs were cultured in 24‐well plates, fixed with 4% paraformaldehyde at 4°C overnight, permeabilized with 0.5% Triton X‐100 for 30 min, and blocked with 10% goat serum for 30 min. For histological staining, the paraffin sections were dehydrated. The tissue sections were stained with hematoxylin and eosin (H&E) (Beyotime Institute of Biotechnology, Shanghai, China) and Masson's trichrome (Servicebio) following the manufacturer's protocols. Primary antibodies used for immunofluorescence analysis included goat anti‐8‐OHdG (Novus, #NB600‐1508), rabbit anti‐TOMM20 (Abcam, #ab186735), F4/80 (D2S9R) XP Rabbit mAb (CST, #70076), and Alexa Fluor 594 anti‐mouse CD206 (MMR) (Biolegend, #141726). DAPI was used for nuclear staining, and images were acquired using a Leica DM6 B Positive Binocular Biomicroscope (Leica).

### 2.10 nDNA and mtDNA Isolation, and Oxidative‐Damaged Mitochondrial DNA Preparation

Nuclei of mouse lungs were isolated using the Nuclear Extraction Kit (Abcam, #ab219177), followed by purification of nDNA with the DNeasy Blood & Tissue Kit (Qiagen, #69504), according to the manufacturer's instructions.

Mitochondria of mice lungs were isolated using the Qproteome mitochondria isolation kit (Qiagen, Germany). mtDNA was isolated using the mitochondria DNA isolation kit (Abcam, USA). The mtDNA was diluted in TE buffer and stored at −20°C for further use. Oxidative‐damaged mitochondrial DNA (oxid‐mtDNA) was obtained by irradiation (25 mJ/cm^2^, 30 min) as previously described [33, 34]. 0.5 µg/U DNase (KeyGEN BioTECH, #KGA1506) was added for DNase‐treated mtDNA or DNase‐treated oxid‐mtDNA.

### 2.11 ELISA Assay

For cytokines in the supernatant of BMDMs, the BMDMs were treated with 20 ng/mL M‐CSF and were added medium (control), 20 µg/mL of BLM, 5 µg/mL of mtDNA, and 5 µg/mL of oxid‐mtDNA separately. The supernatant of BMDMs was collected after 24 h and stored at −20°C. For TGF‐β in BALF, BALF from euthanized mice was centrifuged, and the supernatant was collected. The concentrations of IL‐6, IL‐10, and TGF‐β were measured using the IL‐6 Mouse ELISA Kit, the IL‐10 Mouse ELISA Kit, and the TGF‐β Mouse ELISA Kit (Thermo Fisher Scientific, USA), respectively.

### 2.12 Hydroxyproline

Lung tissues were divided into approximately 0.1 g portions on ice, and the exact weight was recorded. The hydroxyproline concentration in the obtained tissue suspension was analyzed using a hydroxyproline assay kit (Nanjing Jiancheng Bioengineering Institute, China).

### 2.13 Statistical Analyses

The data were analyzed using GraphPad Prism 10. For investigating potential associations between two variables, Student's two‐tailed *t*‐test was employed. Statistical significance was determined by one‐way ANOVA for multiple groups and Student's *t*‐tests for two groups. Data were represented as mean ± SD. The statistical significance was set as *p* < 0.05, *p* < 0.01, *p* < 0.001, *p* < 0.0001 (remarked with *, **, ***, ****).

## 3 Results

### 3.1 BLM‐Induced Lung Inflammation Is Associated With Oxidative Stress

**FIGURE 1:**
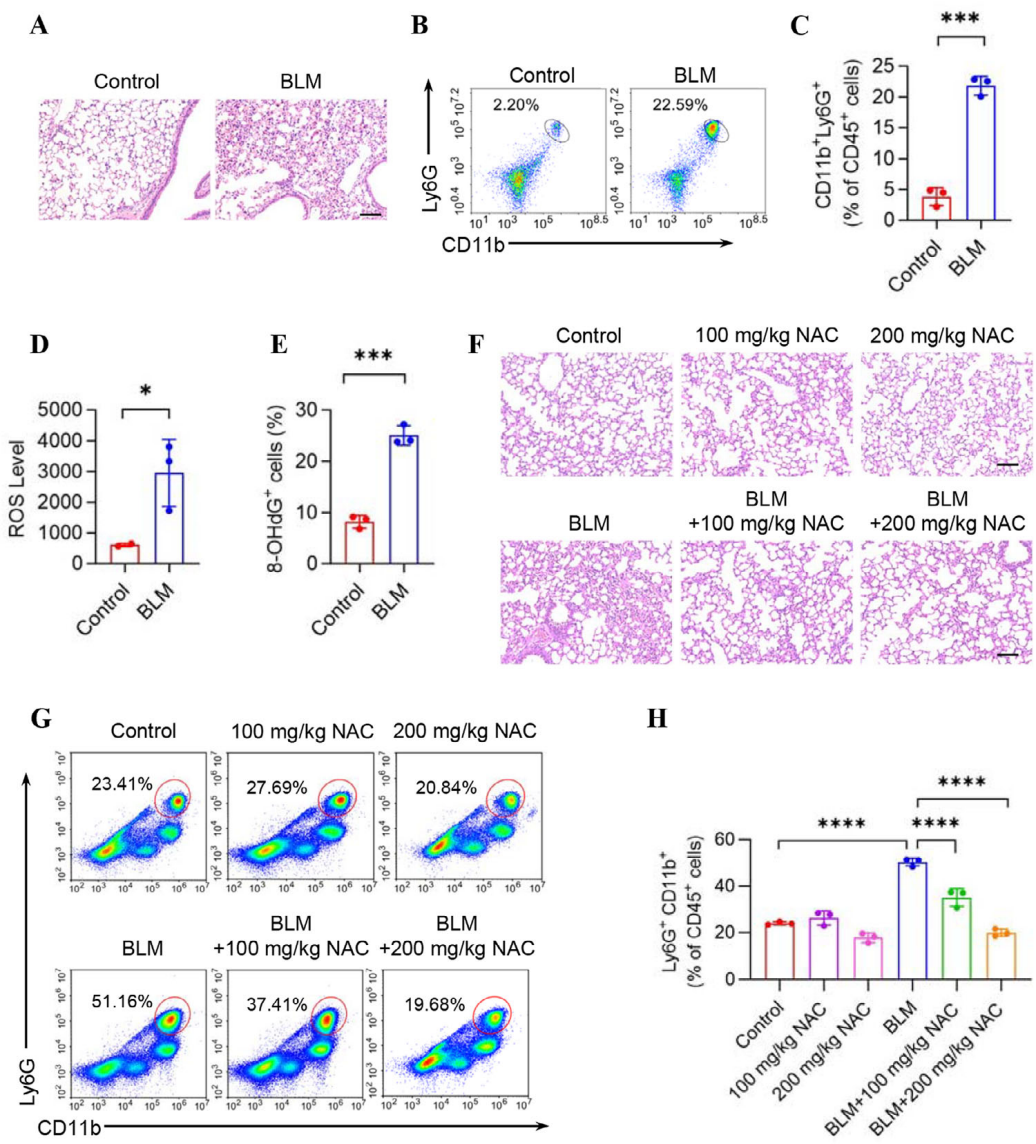
The ROS‐induced oxidative damage caused by bleomycin mediates the pulmonary inflammatory environment. (A–C) Bleomycin (2 mg/kg) was administered intratracheally to C57BL/6 mice. Three days of post‐treatment, the lung tissues were harvested and analyzed by H&E and FCM. H&E staining of lung tissues is shown in (A). Scale bars represent 50 µm. Representative scatterplots of the gated neutrophil (CD45^+^CD11b^+^Ly6G^+^) in the lungs are shown in (B) and quantified in (C) (*n* = 3 mice). (D and E) Bleomycin (20 µg/mL) was added to the culture medium of MPFs. The ROS levels were measured 5 min later (D), and 8‐OHdG levels were detected after 24 h (E) using FCM (*n* = 3 biologically independent samples). (F–H) Mice were intratracheally injected with saline or bleomycin (2 mg/kg). Thirty minutes before the intratracheal injection of bleomycin, mice in the BLM+NAC group or NAC group were intraperitoneally injected with NAC (100 mg/kg or 200 mg/kg, as indicated in group labels). Lung tissues were collected 24 h later. (F) Representative H&E staining of lung tissues. Scale bars represent 50 µm. (G and H) Representative scatterplots with percentages of the gated neutrophils (CD45^+^CD11b^+^Ly6G^+^) in lung tissues are shown in the left panel (G) and quantified in the right panel (I) by FCM analysis (*n* = 3 mice). Data are represented as mean ± SD. Statistical significances in (C–E) were determined by two‐sided unpaired *t*‐test. Statistical significances in (H) were determined by one‐way ANOVA. *p < 0.05, ***p < 0.001, ****p < 0.0001. 8‐OHdG, 8‐hydroxy‐2′‐deoxyguanosine; BLM, bleomycin; FCM, flow cytometry; MPF, mouse pulmonary fibroblast; NAC, N‐acetylcysteine; ROS, reactive oxygen species.

Since pulmonary fibrosis develops from early persistent inflammation, we performed intratracheal injection of BLM in mice to confirm that BLM induces an inflammatory microenvironment in the lungs of mice. Histological changes caused by BLM were assessed by H&E staining, and neutrophil infiltration was measured to evaluate the level of BLM‐triggered inflammation in lungs. In the control group, no significant histological changes or neutrophil infiltration were observed. In contrast, in the BLM group, the alveolar structure became disorganized, and there was an increase in neutrophil infiltration within the alveoli (Figure 1A–C).

Oxidative stress arises from an imbalance between prooxidants and antioxidants [33], leading to reactive oxygen species (ROS) accumulation during inflammation and fibrosis. The lungs are especially susceptible due to high oxygen exposure [34]. ROS can cause DNA oxidation and damage [35], and 8‐OHdG, a byproduct of ROS attack on DNA molecules, is commonly used as a marker of oxidative DNA damage [36]. Elevated ROS and 8‐OHdG levels in mouse pulmonary fibroblasts (MPFs) were observed after treatment with BLM, demonstrating that BLM can induce oxidative stress and DNA damage (Figure 1D,E). Together, these findings revealed that BLM could cause DNA oxidation in MPFs.

To investigate whether inhibiting oxidative stress can alleviate inflammation and neutrophil recruitment, we performed intratracheal injection of BLM alone or with the antioxidant N‐acetylcysteine (NAC) in mice. H&E staining and flow cytometric analyses of lung tissue showed that BLM increased the proportion of neutrophils in the lungs, whereas NAC treatment resulted in a decrease, consistent with the previous pathological results (Figure 1F–H). The antioxidant NAC alleviated BLM‐induced lung inflammation in vivo, suggesting that oxidative stress is a mediator in the inflammatory response triggered by BLM.

### 3.2 Oxidative Damaged‐mtDNA (Oxid‐mtDNA) Released by BLM Treatment Induces Inflammation in Lung

Mitochondria serve as the predominant intracellular site for ROS generation, with oxidative phosphorylation representing the principal source of these reactive molecules. ROS induce oxidative damage to DNA by modifying nucleic acid bases, resulting in the formation of oxidized lesions such as 8‐OHdG [21]. It has been demonstrated that mtDNA can activate neutrophils, thereby inducing inflammation in vivo [39, 40].

**FIGURE 2:**
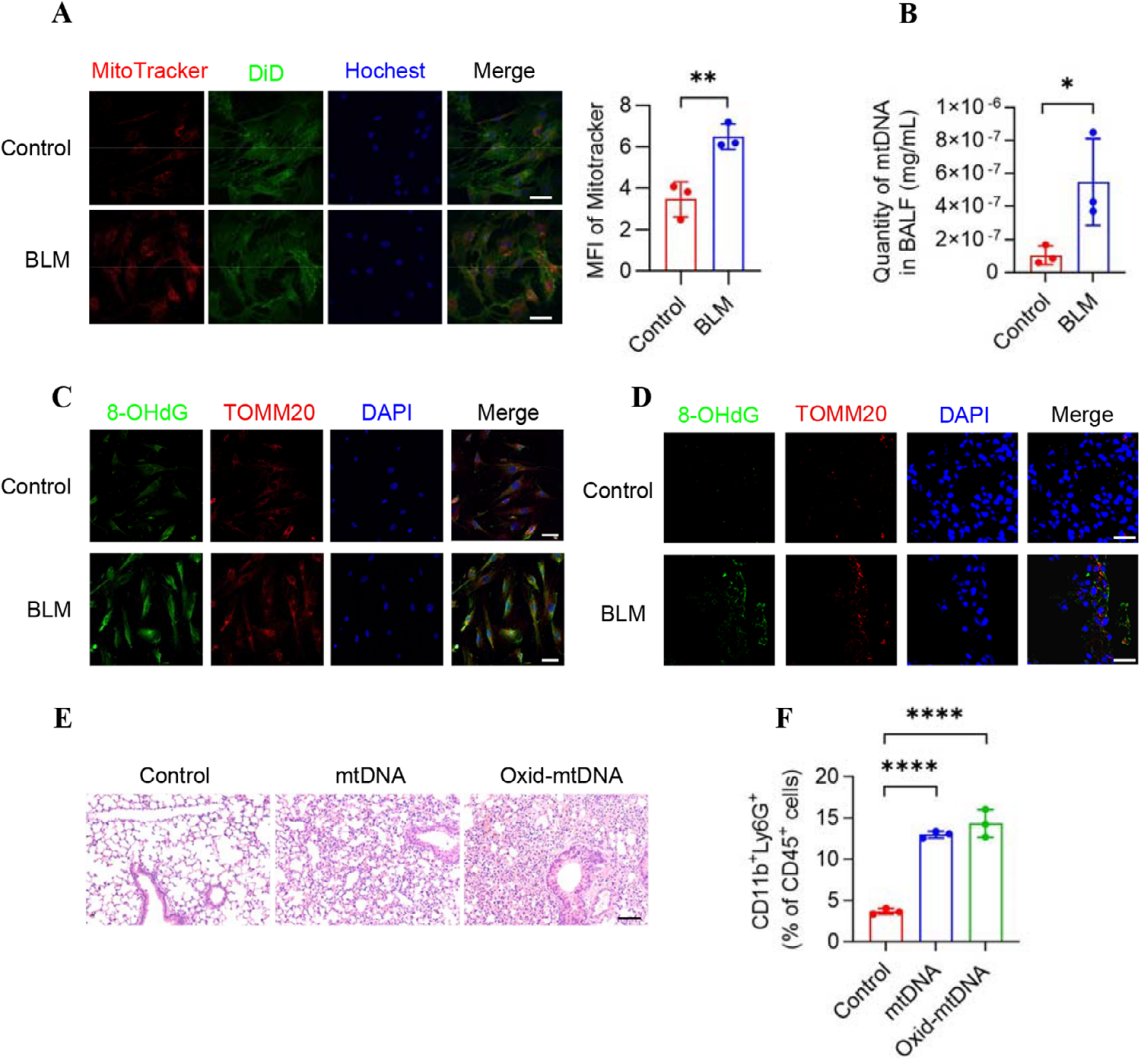
Bleomycin induces oxidative damage to mtDNA and exacerbates inflammation. (A) MPFs were treated with bleomycin (20 µg/mL) and collected 24 h later for immunofluorescence staining with MitoTracker (red), DiD (green, representing cell membrane), and Hoechst (blue) in vitro. Representative immunofluorescence images are shown in the left panel, and quantified MFI of MitoTracker is shown in the right panel (*n* = 3 biologically independent samples). Scale bars represent 100 µm. (B) Bleomycin induced the increase of mtDNA release in vivo. C57BL/6 mice were intratracheally administered with BLM (2 mg/kg), and BALF was collected 24 h later. mtDNA was extracted from the centrifuged supernatant and quantified by qPCR (*n* = 3 mice). (C) MPFs were treated with BLM (20 µg/mL) and collected 24 h later for immunofluorescence staining of 8‐OHdG (green), TOMM20 (red), and DAPI (blue). Scale bars represent 75 µm. D. Mice were intratracheally administered with BLM (2 mg/kg), and lung tissues were collected 24 h later for immunofluorescence staining of 8‐OHdG (green), TOMM20 (red), and DAPI (blue). Scale bars represent 75 µm. (E–F) Mice were intravenously injected with saline, mtDNA (5 µg/each), and oxid‐mtDNA (5 µg/each). After 24 h, lung tissues were collected for H&E staining (E) and FCM analysis to quantify neutrophils (CD45^+^ Ly6G^+^ CD11b^+^) (F). Scale bars represent 50 µm (*n* = 3 mice). Data are represented as mean ± SD. Statistical significances in (A and B) were determined by a two‐sided unpaired *t*‐test. Statistical significances in (F) were determined by one‐way ANOVA. *p < 0.05, **p < 0.01, ****p < 0.0001. 8‐OHdG, 8‐hydroxy‐2′‐deoxyguanosine; BALF, bronchoalveolar lavage fluid; BLM, bleomycin; DAPI, 4',6‐diamidino‐2‐phenylindole; FCM, flow cytometry; MFI, mean fluorescence intensity; MPF, mouse pulmonary fibroblast; mtDNA, mitochondrial DNA; qPCR, quantitative PCR; TOMM20, translocase of the outer mitochondrial membrane 20.

To further explore whether BLM could induce the release of mtDNA, we treated MPFs with BLM in vitro, and an obvious increase of mitochondrial release in BLM‐treated MPFs was observed (Figure 2A). Meanwhile, the level of mtDNA in BALF supernatant from BLM‐treated mice showed a significant increase (Figure 2B). Immunofluorescence staining of both BLM‐treated MPFs and mice showed an increase of 8‐OHdG, which was partially overlapped with TOMM20, suggesting that mtDNA was oxidized (Figure 2C,D).

Moreover, to investigate whether the inflammatory environment in lungs was triggered by oxid‐mtDNA, we extracted and purified mtDNA from mice lung tissues, and prepared oxid‐mtDNA by irradiation. Mice were intravenously injected with saline, mtDNA, or oxid‐mtDNA, respectively. H&E staining suggested that both mtDNA and oxid‐mtDNA caused inflammation in lungs, whereas oxid‐mtDNA induced a more severe inflammation (Figure 2E). Meanwhile, upregulated proportion of neutrophil infiltration in lung tissues from mtDNA and oxid‐mtDNA was observed by flow cytometry (Figure 2F). Therefore, our findings suggest that BLM induces neutrophil infiltration in lung tissues through the mediation of oxidative stress triggered by oxid‐mtDNA.

### 3.3 BLM Induces M2‐Like Polarization of Macrophage Through Mediation by Oxid‐mtDNA

Macrophages play a crucial role in the fibrosis phase of BLM‐induced pulmonary fibrosis, which can be classified into M1 and M2 types based on their response to stimuli. M1 macrophages promote pro‐inflammatory responses by enhancing phagocytosis and antigen presentation, whereas M2 macrophages exert anti‐inflammatory and tissue‐repair functions through the secretion of IL‐10 and TGF‐β [41, 42, 43, 44].

**FIGURE 3:**
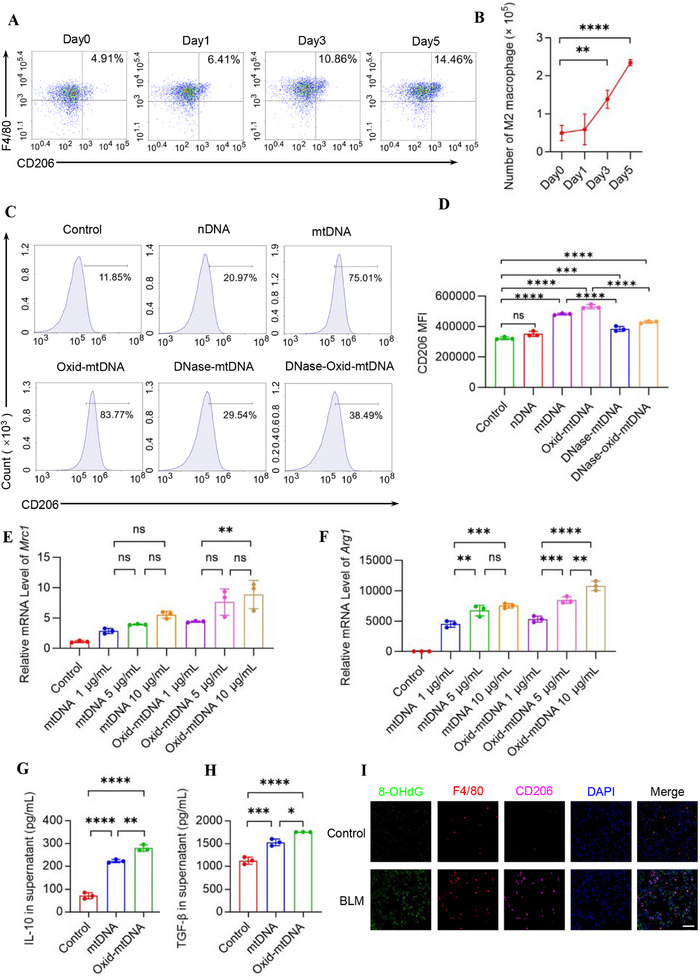
BLM‐induced oxidized mtDNA promotes M2 macrophage polarization. (A and B) Mice were intratracheally administered BLM (2 mg/kg), and lung tissues were harvested at the indicated time points for FCM analysis. Representative scatterplots with percentages of the gated M2 macrophages (CD45^+^ F4/80^+^ CD206^+^ CD11c^−^) are shown in (A) and quantified in (B) (*n* = 3 mice). (C–F) Oxid‐mtDNA promoted M2‐like polarization of BMDMs. BMDMs were derived from C57BL/6 mice and cultured for 4 days. On Day 4, the medium was replaced with fresh culture medium containing 5 µg/mL nDNA, 5 µg/mL DNase‐treated mtDNA, 5 µg/mL DNase‐treated oxid‐mtDNA, mtDNA, or Oxid‐mtDNA (1, 5, or 10 µg/mL as indicated in group labels), and cells were further incubated for 48 h. Cells were then collected for subsequent analyses. Representative histograms of the gated M2 macrophages (CD45^+^CD11c^−^F4/80^+^CD206^+^) are shown in the left panel in (C), and the MFI value is shown in the right panel in (D). Relative expression of CD206 (E) or Arg‐1 (F) in BMDMs was quantified by qRT‐PCR (*n* = 3 biologically independent samples). (G and H) The levels of IL‐10 (G) and TGF‐β (H) in the supernatants of BMDMs from control, mtDNA, and oxid‐mtDNA were detected by ELISA (*n* = 3 biologically independent samples). (I) Mice were intratracheally administered BLM (2 mg/kg), and lung tissues were harvested on Day 21. Immunofluorescence staining of 8‐OHdG (green), F4/80 (red), CD206 (far‐red), and DAPI (blue) in lung tissues of mice. Scale bars represent 50 µm. Data are represented as mean ± SD. Statistical significances in (B) were determined by a two‐sided unpaired *t*‐test. Statistical significances in (D–F and H–K) were determined by one‐way ANOVA. *p < 0.05, **p < 0.01, ***p < 0.001, ****p < 0.0001. BLM, bleomycin; BMDMs, bone marrow‐derived macrophages; ELISA, enzyme‐linked immunosorbent assay; FCM, flow cytometry; MFI, mean fluorescence intensity; mtDNA, mitochondrial DNA; oxid‐mtDNA, oxidative mitochondrial DNA.

To investigate the impact of BLM on pulmonary macrophages, mice were subjected to intratracheal administration of BLM. Flow cytometric analysis of lung macrophages was performed at Days 0, 1, 3, and 5 post‐treatment. The results demonstrated a time‐dependent upregulation of M2 macrophages in BLM‐treated mice (Figure 3A,B). To further investigate the changes of polarization in macrophages, we treated BMDMs with medium, mtDNA, and oxid‐mtDNA separately in vitro. We found that, similar to the control, nDNA did not induce M2 polarization in BMDMs, unlike mtDNA or oxidized mtDNA (Figure 3C,D). DNase treatment markedly reduced the inductive capacity of both mtDNA and oxidized mtDNA, although DNase‐treated oxidized mtDNA retained partial activity compared with the control (Figure 3C,D). These results indicate that both the mitochondrial DNA backbone and its oxidative modifications contribute to M2 polarization, with oxidative modifications exerting a stronger effect. We also conducted dose‐response experiments using mtDNA or oxid‐mtDNA and observed that their ability to induce M2‐like polarization in BMDMs increased progressively with higher doses. However, there was no obvious difference between 5 and 10 µg/mL (Figure 3E,F). Therefore, we selected 5 µg/mL for subsequent experiments. Levels of M2 macrophage‐related cytokines IL‐10 and TGF‐β in the supernatant also showed a more potent response to in vitro stimulation of oxid‐mtDNA (Figure 3G,H). Moreover, we observed colocalization of 8‐OHdG with M2 macrophages in lung tissue (Figure 3I). These findings demonstrate that both mtDNA and oxid‐mtDNA promote M2‐like macrophage polarization, with oxid‐mtDNA exhibiting a more potent effect than mtDNA.

### 3.4 Injection of Oxidized‐mtDNA Induces Pulmonary Fibrosis in Mice

**FIGURE 4:**
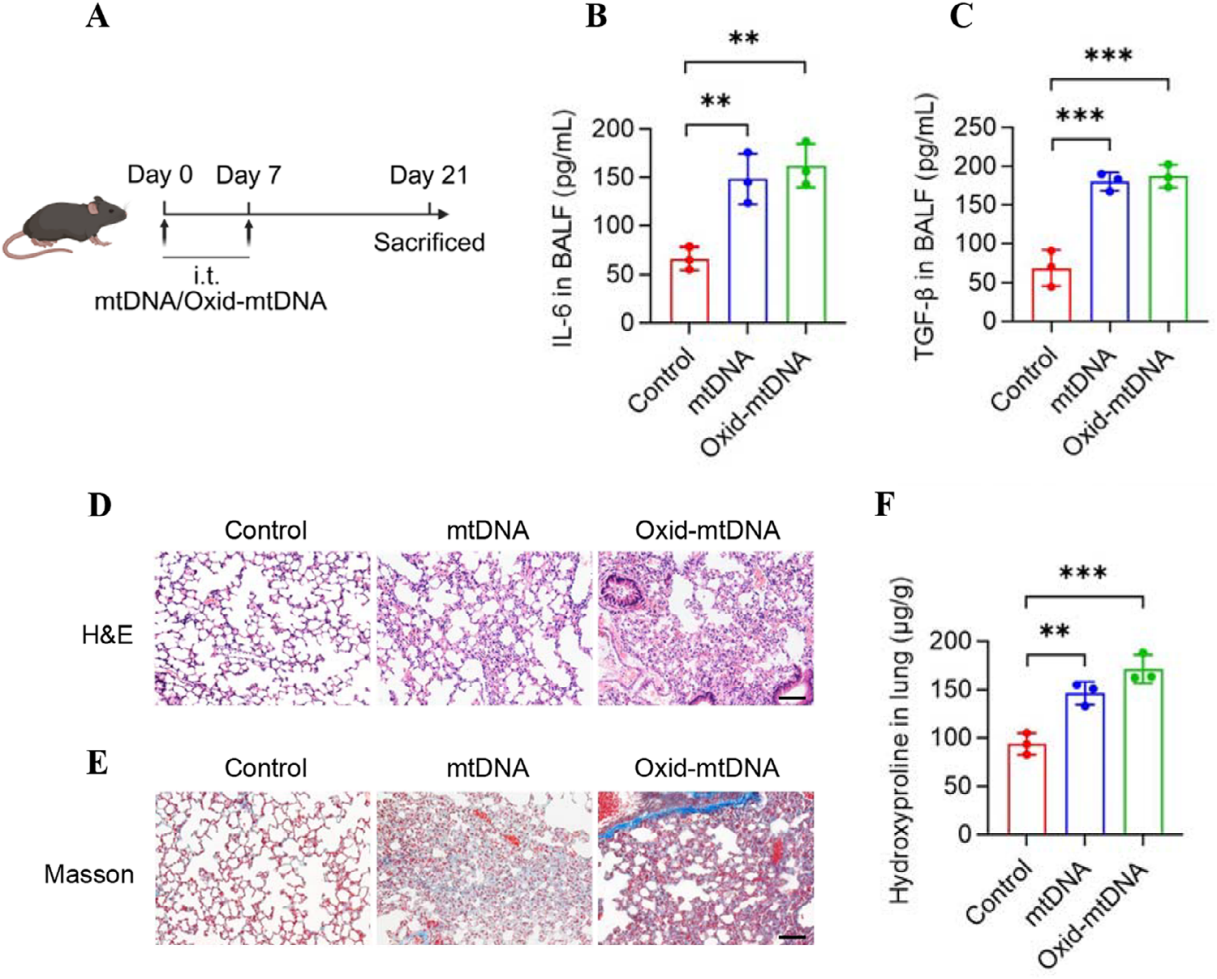
Injection of oxidized mtDNA induces pulmonary fibrosis in mice. (A) Schematic representation of mtDNA‐induced lung fibrosis. Mice were twice intratracheally administered saline, mtDNA (3 µg/each), and oxid‐mtDNA (3 µg/each) on Days 0 and 7. Mice were euthanized, and lung tissues were harvested for further analysis on Day 21 (*n* = 3 mice). (B and C) The levels of IL‐6 (B) and TGF‐β (C) in BALF obtained from mice in A were quantified using ELISA. (D) H&E staining of lung tissues from mice in (A). Scale bars represent 100 µm. (E) Masson staining of lung tissues from mice in (A). Scale bars represent 100 µm. (F) The levels of hydroxyproline in lung tissues of mice from (A). Data are represented as mean ± SD. Statistical significances in (D, E, F, and H) were determined by one‐way ANOVA. **p < 0.01, ***p < 0.001. BALF, bronchoalveolar lavage fluid; ELISA, enzyme‐linked immunosorbent assay; i.t., intratracheal injection; mtDNA, mitochondrial DNA; oxid‐mtDNA, oxidative mitochondrial DNA.

BLM‐induced pulmonary fibrosis is a commonly used model in preclinical pulmonary fibrosis studies. To determine whether BLM‐induced pulmonary fibrosis is mediated by oxid‐mtDNA, mice received intravenous injections of saline, mtDNA, or oxid‐mtDNA on Days 0 and 7 (Figure 4A).

On Day 21, mice were euthanized, and we observed that levels of IL‐6 and TGF‐β from BALF of oxid‐mtDNA‐treated mice were increased (Figure 4B,C). Histopathological analysis of lung tissue via H&E and Masson staining demonstrated that mice treated with oxid‐mtDNA exhibited significantly more pronounced lung damage compared to those treated with mtDNA (Figure 4D,E). Specifically, the oxid‐mtDNA‐treated mice showed increased fibroblast proliferation and collagen deposition, as well as notable thickening of the alveolar septa (Figure 4D,E). We further quantified the hydroxyproline content in lung tissues to determine collagen deposition and found that the hydroxyproline content was higher in the lungs of oxid‐mtDNA‐treated mice than that in mtDNA‐treated mice (Figure 4F). Collectively, we found that oxid‐mtDNA induced significantly more severe pulmonary fibrosis than mtDNA, implicating that BLM induced pulmonary fibrosis through oxid‐mtDNA.

### 3.5 Pulmonary Fibrosis Induced by BLM Is Equally Associated With STING Pathway and NLRP3 Pathway

Given the known association between mtDNA and the cGAS‐STING pathway [45, 46, 47], and the previous findings suggesting a potential link between the STING and NLRP3 signaling pathways [46], we investigated whether the mechanism of BLM‐induced pulmonary fibrosis involves these two classical innate immune pathways. It has been well established that activation of the STING pathway induces the expression of IFN‐β [47], while the release of IL‐18 typically depends on NLRP3 inflammasome activation [48].

**FIGURE 5:**
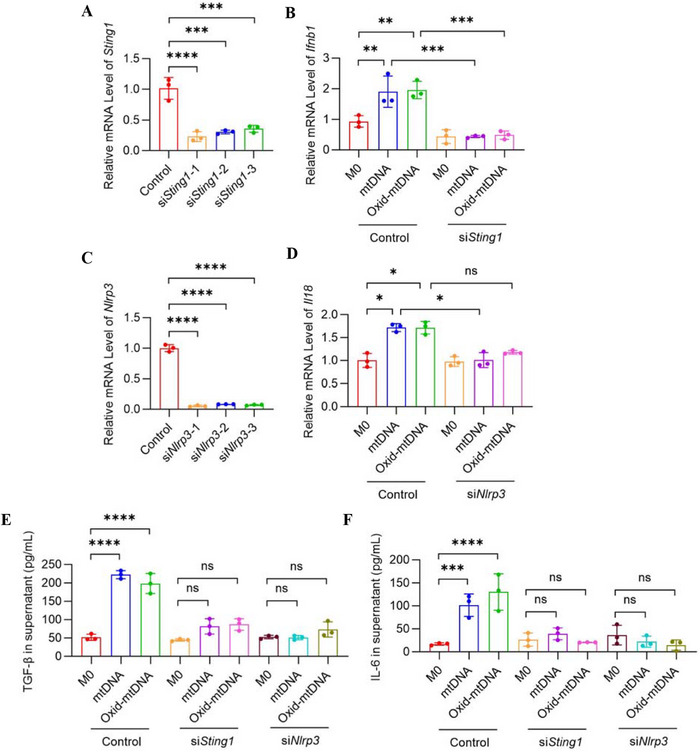
Bleomycin‐induced pulmonary fibrosis is associated with the STING and NLRP3 pathways. BMDMs were isolated from mice and cultured for 3 days. BMDMs were transfected with siRNA on Day 3. After 24 h of siRNA transfection, the medium was replaced with fresh medium containing saline, 5 µg/mL mtDNA, or 5 µg/mL oxid‐mtDNA, and cells were stimulated for an additional 48 h. Cells and supernatants were collected for further analyses. (A and B) Relative expression of *Sting1* (A) and *Ifnb1* (B) in BMDMs were quantified by qRT‐PCR (*n* = 3 biologically independent samples). (C and D) Relative expression of *Nlrp3* (C) and *Il18* (D) in BMDMs were quantified by qRT‐PCR (*n* = 3 biologically independent samples). (E and F) The levels of TGF‐β (E) and IL‐6 (F) in the supernatants were detected by ELISA (*n* = 3 biologically independent samples). Data are represented as mean ± SD. Statistical significances in (A–F) were determined by one‐way ANOVA. ns, no significance; *p < 0.05, **p < 0.01, ***p < 0.001, ****p < 0.0001. BLM, bleomycin; BMDMs, bone marrow‐derived macrophages; mtDNA, mitochondrial DNA; NLRP3, NOD‐like receptor pyrin domain‐containing protein 3; oxid‐mtDNA, oxidative mitochondrial DNA; qRT‐PCR, quantitative real‐time PCR; siRNA, small interfering RNA; STING, stimulator of interferon genes; WT, wild type.

To explore this, we used small interfering RNA (siRNA) to knock down STING expression in BMDMs isolated from wild‐type (WT) mice. Stimulation with mtDNA or oxid‐mtDNA upregulated downstream markers of the STING and NLRP3 pathways in WT BMDMs, with oxid‐mtDNA showing stronger effects. siSting1 suppressed STING1 and its downstream marker Ifnb1, while siNlrp3 reduced Nlrp3 and Il18 expression (Figure 5A–D). Collectively, we found that downstream markers of STING and NLRP3 pathways were inhibited in a genotype‐dependent manner. Furthermore, we examined cytokine release in the culture supernatant. We found that siRNA suppressed the cellular response of TGF‐β and IL‐6 release to mtDNA or oxid‐mtDNA (Figure 5E,F). The results indicate that WT versus siRNA‐treated cells respond differently to in vitro stimulation with mtDNA or oxid‐mtDNA.

STING and NLRP3 are both signaling pathways closely related to mtDNA that can be activated by mtDNA or oxid‐mtDNA, initiating downstream cascades that promote the secretion of pro‐inflammatory cytokines, including IL‐6, IL‐10, and TGF‐β, ultimately contributing to the progression of pulmonary fibrosis [51, 52, 53, 54].

**FIGURE 6:**
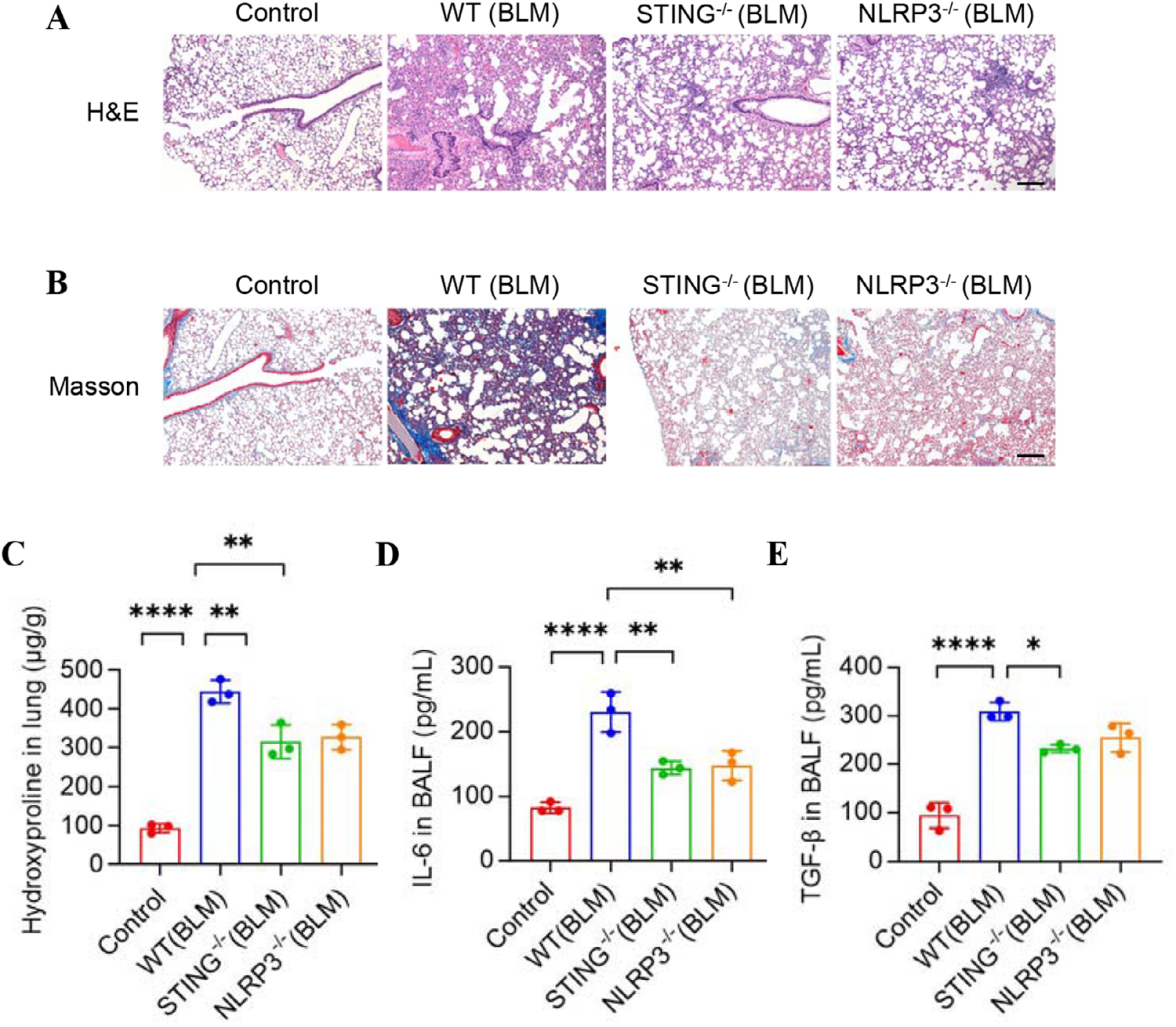
STING or NLRP3 deficiency attenuates bleomycin‐induced pulmonary fibrosis in vivo. BLM (2 mg/kg) was administered intratracheally to WT, STING‐/‐, and NLRP3‐/‐ mice, and lung tissues were collected on Day 21 (*n* = 3 mice). (A and B) H&E and Masson staining of lung tissues. Scale bars represent 100 µm. (C) The levels of hydroxyproline in lung tissues from mice in (A). (D and E) The levels of IL‐6 (I) and TGF‐β (J) in BALF of mice were quantified by ELISA. Data are represented as mean ± SD. Statistical significances in (C–E) were determined by one‐way ANOVA. *p < 0.05, **p < 0.01, ****p < 0.0001. BALF, bronchoalveolar lavage fluid; BLM, bleomycin; ELISA, enzyme‐linked immunosorbent assay; NLRP3, NOD‐like receptor pyrin domain‐containing protein 3; STING: stimulator of interferon genes.

To further investigate whether BLM‐induced pulmonary fibrosis is related to STING or NLRP3 signaling pathways, we established BLM‐induced pulmonary fibrosis models in WT mice, STING^−/−^ mice, and NLRP3^−/−^ mice. Fibrotic pathology was evident in H&E staining of lungs from WT mice, characterized by alveolar disruption, septal thickening, and pronounced pulmonary fibrosis. In contrast, STING^−/−^ and NLRP3^−/−^ mice exhibited markedly attenuated fibrotic progression (Figure 6A). Masson staining further confirmed substantial collagen deposition and architectural distortion in lungs from WT mice, whereas STING^−/−^ and NLRP3^−/−^ mice demonstrated significantly reduced collagen accumulation and milder fibrotic remodeling (Figure 6B). Moreover, hydroxyproline content in lung tissue, used as an indicator of collagen deposition, was significantly reduced in STING^−/−^ mice and NLRP3^−/−^ mice (Figure 6C). Consistently, levels of inflammatory cytokines IL‐6 and TGF‐β in BALF were also decreased in these knockout mice, which was consistent with the pathological findings (Figure 6D,E). Therefore, these findings suggest that BLM‐induced pulmonary fibrosis is dependent on the activation of the STING and NLRP3 signaling pathways.

## 4 Discussion

Our primary finding was that oxid‐mtDNA plays a pivotal role in the initiation of lung inflammation and the progression of the disease into pulmonary fibrosis. Additionally, it was discovered that the STING signaling pathway actively participates in oxid‐mtDNA‐mediated processes under these conditions. We also highlighted the intriguing possibility of developing therapies targeting the oxid‐mtDNA‐STING signaling pathway for the prevention and treatment of lung inflammation and pulmonary fibrosis.

BLM is a potent antitumor agent with serious dose‐limiting side effects such as pulmonary toxicity (e.g., mesenchymal fibrosis) [53]. However, the mechanism of BLM‐induced pulmonary fibrosis remains incompletely understood. Oxidative stress is closely linked to pulmonary fibrosis, as evidenced by the presence of ROS in patients and animal models of pulmonary fibrosis.

We first demonstrated that BLM can induce an inflammatory response in mice and promote the infiltration of neutrophils into lung tissue. Subsequently, we used MPFs in vitro to show that BLM treatment increases the release of ROS. Since ROS have been reported to cause oxidative damage to DNA, we demonstrated that BLM can induce oxidative damage to intracellular DNA through ROS. We treated mice with the antioxidant NAC, which partially mitigated the BLM‐induced inflammation, indicating that the inflammatory response induced by BLM is dependent on oxidative stress.

Next, we observed that BLM induces the release of mtDNA both in vivo and in vitro, and found that both mtDNA and oxid‐mtDNA can induce lung inflammation in mice, with oxid‐mtDNA demonstrating a more potent effect. Given the critical role of M2 macrophages in the inflammatory and fibrotic processes, we confirmed that M2 macrophages accumulate in the inflammatory environment of lungs induced by BLM. Furthermore, we verified that oxid‐mtDNA can induce M2‐like polarization of macrophages using BMDMs and PMs in vitro.

We then attempted to use oxid‐mtDNA to induce pulmonary fibrosis in mice, resulting in severe fibrosis in the lung tissues of oxid‐mtDNA‐treated mice. It is worth noting that mtDNA and oxid‐mtDNA increased the production of TGF‐β, IL‐6, and IL‐10 by BMDMs. TGF‐β is a known factor critical for fibrosis progression, and TGF‐β expression is increased in several fibrotic diseases and animal models [56, 57, 58, 59]. Hence, we next examined some of the important signaling pathways through which TGF‐β and oxidative stress work together to drive lung fibrosis progression. Finally, we discovered that STING^−/−^ mice and NLRP3^−/−^ mice exhibited protective effects against BLM‐induced pulmonary fibrosis, indicating that BLM‐induced pulmonary fibrosis is dependent on these two signaling pathways.

Mitochondrial components and metabolites, such as N‐formyl peptides, participate in the regulation of immune responses through DAMPs, influencing neutrophils, macrophages, and other immune‐associated cells [47, 60, 61, 62, 63]. In this study, we found that BLM induced the release of mitochondria and mtDNA. Using co‐immunofluorescence staining techniques, we discovered that BLM‐induced mtDNA release led to oxidative damage both in vivo and in vitro. Additionally, it was demonstrated that both mtDNA and oxid‐mtDNA induced lung inflammation, with oxid‐mtDNA exhibiting a more potent effect, resulting in a higher magnitude of neutrophil infiltration in lung tissue. These results suggest that BLM‐induced ox‐mtDNA release led to neutrophil recruitment in lung tissues and increased oxidative stress. To further elucidate the underlying mechanisms, we demonstrated that oxid‐mtDNA directly induced pulmonary fibrosis in mice and stimulated fibroblast proliferation through macrophages.

We propose that the STING and NLRP3 pathways play a synergistic role in the mechanism of BLM‐induced pulmonary fibrosis. BLM induces mitochondrial oxidative stress, leading to the production of ROS and the release of mtDNA; thus, the cGAS‐STING and NLRP3 pathways share upstream regulatory signals [64, 65]. cGAS‐STING and NLRP3‐IL‐1β pathways amplify and compensate for each other. STING recruits and further activates NLRP3 to promote inflammasome assembly [66, 67]. Proinflammatory cytokines from the NLRP3‐IL‐1β pathway, such as IL‐1β, also increase Sting1 expression, thereby upregulating cGAS‐STING to amplify DNA‐sensing signals [66]. Importantly, we found that two mtDNA‐involved signaling pathways, STING and NLRP3 signaling, are directly involved in BLM‐induced pulmonary fibrosis. These findings reveal for the first time the critical roles of STING and NLRP3 in the progression of BLM‐induced pulmonary fibrosis.

BLM is widely used to model pulmonary fibrosis, yet its mechanisms remain unclear. Our study identifies a novel role for BLM‐induced oxid‐mtDNA, which simultaneously activates the STING and NLRP3 pathways to drive fibrotic responses.

Based on STING and NLRP3 pathways, current strategies targeting oxid‐mtDNA mainly involve modulating the cytosolic DNA sensor cGAS or reducing mtDNA oxidative stress. Aberrant activation of the cGAS‐STING axis during lung injury can promote immune‐cell recruitment and fibroblast activation, making this pathway an attractive therapeutic target [67]. However, dedicated clinical data on pulmonary fibrosis remain lacking [68]. Thus, cGAS inhibition, while promising, still requires further mechanistic and safety evaluation. Mitochondrial ROS and mtDNA damage activate NLRP3 and TGF‐β signaling, amplifying profibrotic responses [69]. The mitochondria‐targeted antioxidant MitoQ reduces mitochondrial ROS and disrupts early fibrotic triggers, though clinical evidence in IPF is still limited [70].

However, this study has several limitations that should be acknowledged. Although we demonstrate that oxidized mtDNA promotes M2 macrophage polarization, the upstream sensing mechanisms, particularly the involvement of cGAS‐STING and NLRP3 signaling, were not fully delineated. As a result, the relative contribution and potential crosstalk among these pathways remain to be clarified. Furthermore, the BLM‐induced fibrosis model primarily reflects an acute injury‐driven process, which differs from the chronic, progressive fibrotic phenotype observed in idiopathic pulmonary fibrosis. This discrepancy may limit the extent to which our findings can be directly extrapolated to human disease.

In conclusion, this study employed both in vitro and in vivo approaches to investigate an important side effect associated with BLM treatment, a commonly used anticancer therapy. The study explored the underlying mechanisms of BLM‐induced lung inflammation and pulmonary fibrosis, which are known to have elusive mechanisms. Our results demonstrated the critical role of mtDNA and oxid‐mtDNA in initiating early‐phase lung inflammation and progressing to pulmonary fibrosis through oxidative stress, together with the STING pathway and NLRP3 pathways. These findings suggest an intriguing role of mtDNA‐related molecular events in chemotherapy‐induced lung injury and highlight the potential of targeting this molecular mechanism for the development of novel treatments for lung inflammation and pulmonary fibrosis.

## Author Contributions

Xiawei Wei and Min Luo conceived the project, designed the experiments, and revised the manuscript. Ye Mao and Xinyu Tian performed experiments, analyzed data, and wrote the manuscript. They contribute equally to the work. Jiayuan Ai and Dandan Wan assisted with language editing, and Xiaoting Zhou and Yanghong Ni contributed to figure revision. All authors read and approved the final manuscript.

## Funding

This work was supported by the National Key Research and Development Program of China (2024YFC2310700, X.W.), 1.3.5 project for disciplines of excellence–Clinical Research Fund, West China Hospital, Sichuan University (ZYGD23038, X.W.), the National Natural Science Foundation of China (82273297, 82573654, M.L.; 82403281, D.W.), and the Natural Science Foundation of Sichuan Province (2024NSFSC1199, D.W.).

## Ethics Statement

All animal studies carried out were approved by the Animal Care and Use Committee of Sichuan University (ethical approval number: 20230307058).

## Conflicts of Interest

The authors declare no conflicts of interest.

## Data Availability

Data will be made available on request.
